# Laparoscopic Correction of Isthmocele and Cesarean Scar Endometriosis: A Report of a Successful Pregnancy and Treatment of Subfertility

**DOI:** 10.7759/cureus.54576

**Published:** 2024-02-20

**Authors:** Jijisha Ali, Gazala Khan, Yevginiy Karamurzin, Rida Maryum, Sami Talo

**Affiliations:** 1 Obstetrics and Gynaecology, Mediclinic Welcare Hospital, Dubai, ARE; 2 Pathology and Laboratory Medicine, Mediclinic City Hospital, Dubai, ARE; 3 College of Medicine, Mohammed Bin Rashid University of Medicine and Health Sciences, Dubai, ARE

**Keywords:** dysmenorrhea, cesarean scar endometriosis, laparoscopy, subfertility, cesarean scar defect

## Abstract

We present a case of subfertility due to isthmocele and cesarean scar endometriosis with a successful pregnancy following laparoscopic repair. This case report is of a 35-year-old female (para 1, living 1) who presented to the gynecological outpatient department with complaints of lower abdominal pain, irregular vaginal bleeding for three months, and subfertility. She was suspected to have isthmocele and endometriosis at the site of the cesarean scar with seroma formation. She underwent a hysteroscopy and laparoscopic excision of the cyst at the site of the cesarean scar with the repair of the cesarean scar defect. Diagnosis of scar endometriosis was confirmed on histopathology. She successfully became pregnant after one year and had a full-term pregnancy and delivered via cesarean section. Cesarean scar defect, also known as isthmocele, emerges as a notable complication following cesarean delivery, often linked with secondary infertility. Other associated complications of scar defect are prolonged menstrual bleeding, dysmenorrhea, dyspareunia, and chronic pelvic pain. The laparoscopic reparation of the uterine scar defect proves to be a successful approach in addressing secondary infertility and subfertility issues. Individuals with a prior cesarean section history, expressing concerns about secondary infertility and distressing complaints, require a thorough examination of the uterine scar before embarking on future pregnancy plans. Scar endometriosis is an uncommon medical condition and can worsen patient symptoms and lead to further complications. Diagnosis is often established following the excision of the lesion and subsequent histopathological examination. Prompt management can relieve patient symptoms and prevent further complications.

## Introduction

Over the past few years, there has been a global rise in the prevalence of cesarean sections, accompanying an escalation in associated risks of complications. The current prevalence of cesarean section varies between 6% and 27.2% from the available data collected [1]. Patients with cesarean scar defects (CSD) are known to have serious gynecological and obstetrical problems [2].

A CSD usually emerges on the anterior wall of the uterine isthmus. Inadequate healing of the cesarean incision leads to the thinning of the anterior uterine wall and as a result, leads to the formation of an indentation and a fluid-filled pouch at the site of the scar [3]. This complication is also known by various terms, including uterine scar defect, cesarean scar syndrome, diverticulum, sacculation, isthmocele, scar pouch, or niche. The choice of surgical technique employed for uterine closure has been identified as a significant determinant in the development of CSD [4]. Some other proposed factors for scar defect include prolonged labor, cervical dilatation of more than 5 cm before cesarean section, the use of oxytocin, a retroverted uterus, and a low incision of the uterus have also been proposed as contributors to the abnormal healing of the cesarean scar [5].

Scar endometriosis is a seldom-occurring medical condition that poses challenges in diagnosis [6]. Typically, the diagnosis is confirmed only following the excision of the lesion and subsequent histopathological examination. Instances of scar endometriosis on the abdominal wall have been documented after various obstetric and gynecological procedures. Nevertheless, the occurrence of endometriosis within a uterine scar is exceptionally rare. The endometrial tissue gets introduced directly into the incision site when a cesarean section is done. Under suitable conditions of nutrient availability and hormonal stimulation, these endometrial cells endure and undergo proliferation, ultimately resulting in cesarean scar endometriosis (CSE). Despite being a rare condition, the proposed incidence of this condition is 0.03-0.45% [7]. CSE and scar defects can lead to persistent discomfort characterized by cyclic lower abdominal pain and secondary infertility. However, after undergoing laparoscopic treatment of the scar defect and excision of the endometrial tissue, 92% of women achieved successful pregnancies [8].

## Case presentation

A 35-year-old female (para 1, living 1) presented at our gynecology clinic with a three-month history of lower abdominal pain and vaginal bleeding on and off. She reported regular menstrual periods but complained of intermenstrual bleeding persisting until the next menstrual cycle. Additionally, she expressed pain during her menstrual period and difficulty conceiving for the past three years. Her last pregnancy ended in an emergency cesarean section due to a lack of progress in labor and failed induction. She also experienced postoperative fever. After initially presenting with these symptoms at another facility, appropriate imaging, including magnetic resonance imaging (MRI), revealed cesarean section scar endometriosis with a seroma. The patient sought further management at our hospital.

**Figure 1:**
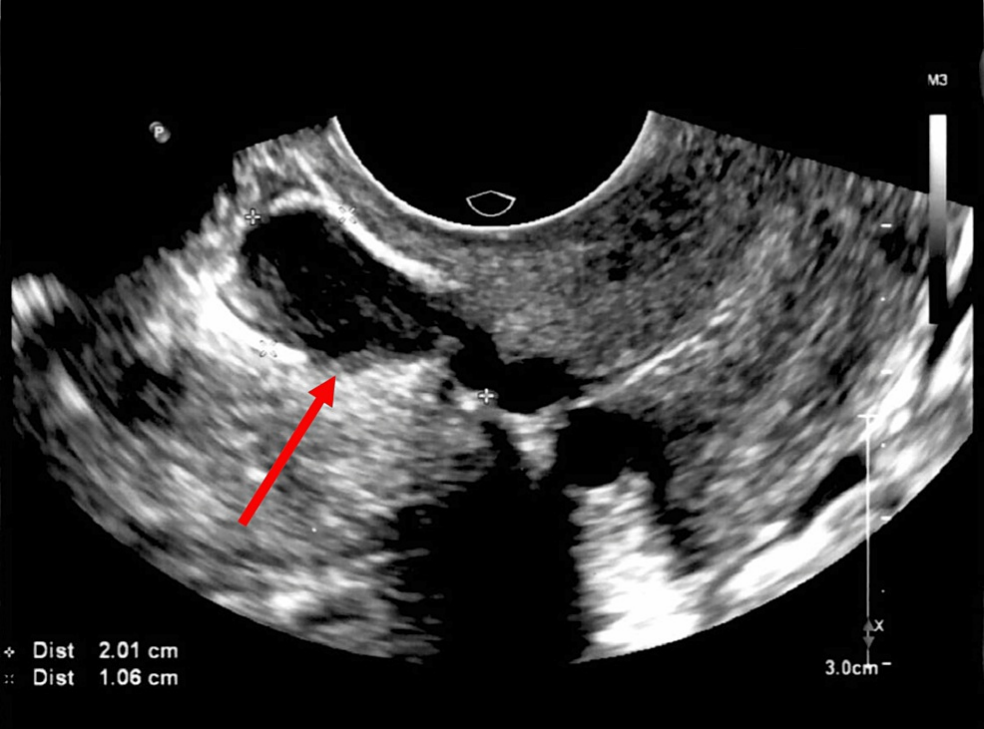
Transvaginal ultrasound showing cesarean scar defect with seroma and scar endometriosis

A transvaginal ultrasound identified a hypoechoic mass of 2.01 x 1.06 cm, with non-union of the myometrial wall as seen in Figure 1. This finding was located at the isthmic region at the level of the cesarean scar, suggesting scar endometriosis and non-union or disruption of the lower uterine segment cesarean section scar. The diagnosis of isthmocele and cesarean section scar endometriosis with cyst was made.

**Figure 2:**
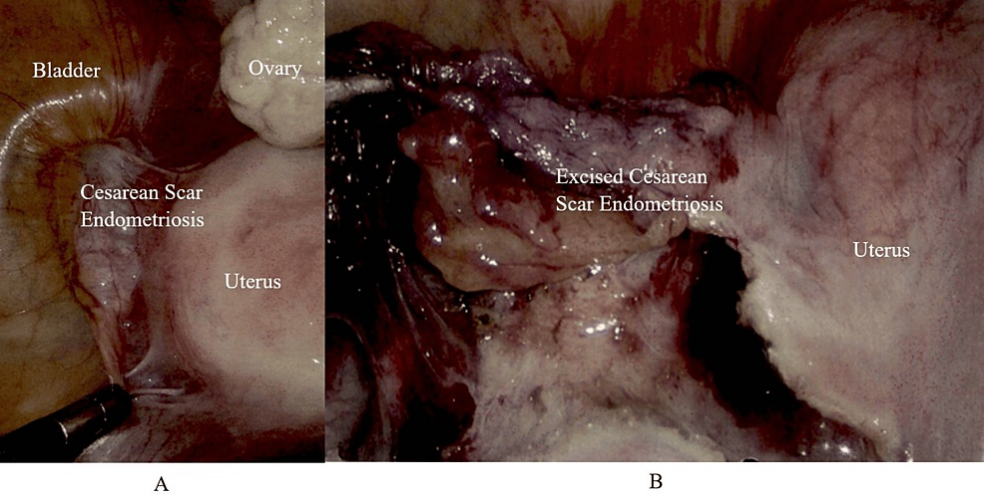
Laparoscopic view showing (A) cesarean scar endometriosis prior to laparoscopic excision and (B) the excised cesarean scar endometriosis

Laparoscopic excision of the cyst at the cesarean scar and excision and repair of the CSD were performed. Initially, a hysteroscopy was performed and a 1 x 1 cm defect was found in the previous cesarean scar noted at the 9 o’clock and 3 o’clock positions. The uterine cavity and both ostia appeared normal. Laparoscopy revealed a bulge inferior to the bladder at the level of the cesarean scar. The bladder was dissected gently from the cyst. The cyst contained fat-like material. The cyst had a connection to the CSD on the right and the left side, as seen in Figure 2A. The bladder adhesions were completely released from the previous cesarean scar site and the cyst material was completely drained out. The defective cesarean scar tissue was excised, as seen in Figure 2B. After the excision of the cesarean scar, a uterine sound was kept inside the uterus, the edges of the scar were sutured in two layers with V-Loc™ wound closure device (Medtronic plc, Dublin, Ireland), and the angles were sutured with PDS™ (Ethicon, Inc. Raritan, New Jersey, United States). At the end of the procedure, a cystoscopy was done. The bladder appeared normal and urine efflux was noted from both ureteric orifices.

**Figure 3:**
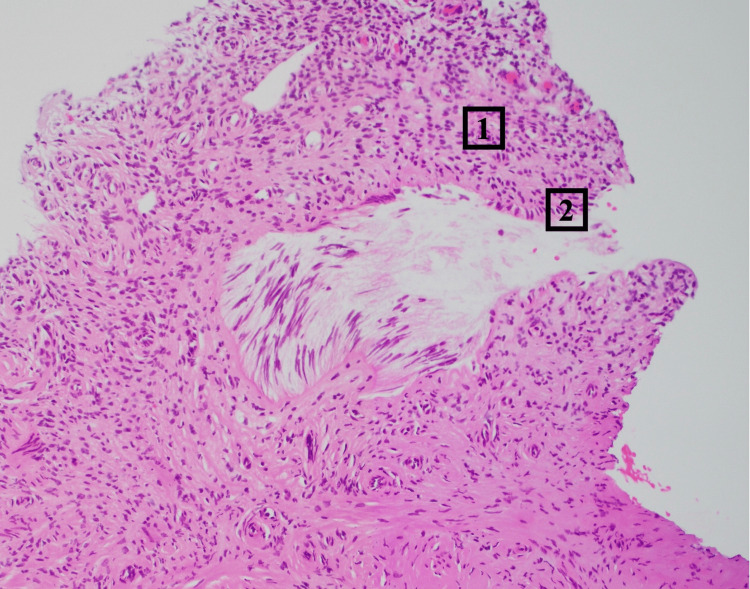
Histopathology slide of the cesarean scar tissue showing endometrial stroma (1) and endometrial glands (2)

The histopathology of the cheesy material from the cyst in the cesarean scar showed benign fibro-adipose and smooth muscle tissue with focal xanthomatous inflammation. The histopathology of the scar tissue showed focal endometriosis as seen in Figure 3.

After surgery, the patient conceived spontaneously after 12 months. She had an uneventful antenatal period until 37 weeks when she was admitted with vaginal bleeding and labor pains. She underwent an emergency caesarian section under spinal anesthesia. Intra-operatively, there was scar dehiscence at the middle of the lower uterine segment scar, and the baby's hair could be seen under the peritoneum. A male baby weighing 2970 grams was born, with an Apgar score of 9 out of 10. Uterine closure was done in two layers and was peritonised. Her postoperative period was uneventful, and she was discharged in satisfactory condition.

## Discussion

As per the available data, there is a lack of agreement on the preferred surgical approach for treating an isthmocele. Before treatment, two key factors are considered when choosing the optimal repair method. These are the size of the isthmocele and the patient's fertility preferences.

Marotta et al. proposed a classification. As per the classification, a large defect is defined as residual myometrium greater than 3 mm, and a small defect is defined as less than 3 mm [9]. Hysteroscopic resection is a preferred, safe, and efficient method for patients with small defects not seeking fertility. Hysteroscopy causes an increased risk of complications such as uterine perforation and bladder injuries when done for defects greater than 3 mm. Consequently, laparoscopy is considered the optimal approach for large defects and for those planning future pregnancies [10].

Donnez et al. studied 38 patients with an isthmocele. The study showed good outcomes postoperatively with no complications and a pregnancy rate of 44% [11]. Morris was the first gynecologist who pioneered the description of uterine scar defects in women following cesarean section. He conducted a detailed analysis of the anatomical and histological abnormalities and alterations present in the scar tissue at the isthmus of the anterior uterine wall [12].

Various factors that may contribute to a uterine scar defect include the use of a single-layer closure for myometrial suturing, multiple cesarean sections, a retroflexed uterus, and an incision present in the cervical area. A study done by Bij de Vaate et al. proposed that women with preeclampsia tend to exhibit a weaker formation of scars [13]. The most common complaint in patients is postmenstrual spotting. Uncommon manifestations include chronic pelvic pain, dysmenorrhea, dyspareunia, and infertility. The severity of these symptoms is directly linked to the size of the uterine scar defect. Women with small defects may exhibit no symptoms, while women with larger scars commonly report extended bleeding durations and are more prone to experiencing a combination of symptoms.

In women with a CSD, there are numerous visualization techniques, such as hysterography, sonohysterography, and transvaginal ultrasound to assess the integrity of the anterior uterine wall. However, the initial diagnostic approach is ultrasound examination. The uterine scar defect is recognized on ultrasound as a "niche", indicated by a triangular hypo-echogenic zone in the uterine scar region [13]. The apex of this triangular zone points towards the anterior uterine wall and the base points towards the uterine cavity or cervical canal. Patients with symptoms should be offered surgical management of the CSD before their next pregnancy [14]. Research findings by Murat et al. indicated that laparoscopic repair of the CSD exhibits greater efficacy when compared to hysteroscopy in preventing abnormally invasive placenta, scar dehiscence, and cesarean scar pregnancies [15].

In our case, the patient complained of prolonged intermenstrual bleeding and lower abdominal pain. She was diagnosed with isthmocele with seroma and cesarean section scar endometriosis. The incidence of CSE is rare (0.03-0.45%) and patients usually complain of cyclic lower abdominal pain. The theory explaining the pathophysiology ofCSE is iatrogenic cell implantation in the wound during surgery [16].

CSE has the potential to undergo malignant transformation, resulting in a rapidly fatal condition with a survival rate as low as 57% [17]. Consequently, it is imperative to implement precautions aimed at preventing or minimizing the occurrence of CSE. The utilization of MRI proved highly beneficial in diagnosing endometriosis. This was attributed to the distinctive signal characteristics observed across different sequences, effectively signaling the presence of blood products within the uterine lesion. In our case, the imaging picked up the scar endometriosis earlier.

A rare case of endometriotic cyst at the CSD was reported by Xu et al., which is similar to our patient’s case [18]. Endometriotic cysts occurring at the site of CSD are an uncommon occurrence. The presence of a pelvic mass within the area, with or without symptoms like menstrual changes and intermittent abdominal pain, should raise the suspicion for this diagnosis. Opting for surgical intervention proves to be a suitable course of action for addressing this condition.

In our case, the endometriotic lesions with the scar defect were completely resected intraoperatively, and the patient was followed up with no significant recurrence and she conceived spontaneously within one year of the procedure. The patient was followed up to date and she has had no recurrence of the disease.

A review of the literature suggests that if a patient intending to conceive exhibits symptoms suggestive of uterine scar insufficiency and has a residual myometrial thickness exceeding 3 mm, it is recommended to undergo regular ultrasound monitoring of the lower uterine segment. Conversely, if the residual myometrium measures below 3 mm, the suggested course of action involves planning a laparoscopy for the resection of scar tissue and the renewal of scar edges [19]. For individuals expressing concerns but not actively seeking pregnancy, the advised approach is operative hysteroscopy. In cases where a scar defect is coincidentally diagnosed without concurrent symptoms, and the patient remains asymptomatic, surgical intervention is not necessary. Following uterine scar repair, it is recommended to conduct routine ultrasound monitoring of the lower uterine segment every month from the 20th to the 32nd week of pregnancy, followed by weekly monitoring until delivery [20]. Delivery is advised at 38-39 weeks of gestation through a planned cesarean section. In situations involving premature delivery and unsuccessful tocolysis, an urgent cesarean section is recommended [20].

Surgical excision of the CSE provides the most reliable opportunity for establishing a conclusive diagnosis and addressing the disease compared to medical therapy, which has a limited success rate and is linked to potential adverse effects.

## Conclusions

The presence of an endometriotic cyst in a CSD is a rare finding and is a significant intraoperative discovery in individuals exhibiting symptomatic isthmocele. Ultrasound investigation is done prior to surgical intervention, with MRI being considered to aid in confirming the diagnosis. Patients with this condition often experience periodic abdominal pain and secondary infertility. Therefore, prompt diagnosis and management are vital to resolve these symptoms and prevent further complications. Opting for the laparoscopic mode of management proves to be both safe and effective. This method is especially recommended for women experiencing secondary infertility, and a preoperative discussion with the patient should address the consideration of simultaneous endometriosis resection. Postoperative pregnancies require vigilant monitoring during follow-up to assess for potential recurrence of the disease.
